# Social Network Exposure to Commercial Sexual Exploitation and Risk of Harm to Youths

**DOI:** 10.1001/jamanetworkopen.2025.13520

**Published:** 2025-06-10

**Authors:** Ieke de Vries, Matthew Kafafian, Sheelah Gobar, Amy Farrell

**Affiliations:** 1 Institute of Criminal Law and Criminology, Leiden University, Leiden, the Netherlands; 2 School of Criminology and Criminal Justice, University of Nebraska, Omaha; 3 Children’s Advocacy Center of Suffolk County, Boston, Massachusetts; 4 School of Criminology and Criminal Justice, Northeastern University, Boston, Massachusetts

## Abstract

**Importance:**

Commercial sexual exploitation (CSE) of young people (aged 6-26 years) poses a considerable public health and social challenge. Understanding how social network exposure to CSE influences CSE risk may inform targeted prevention strategies for at-risk youths.

**Objective:**

To examine the association between social network exposure to CSE and personal risk of experiencing CSE among youths, with a focus on how exposure interacts with childhood adversities.

**Design, Setting, and Participants:**

This cross-sectional study used data collected between January 1, 2015, and December 31, 2022, from a CSE program at a children’s advocacy center in the northeastern US. Eligibility criteria included referral for suspected or confirmed CSE risk. Social relationships with offenders, peers, and others sharing residential placements were examined as potential sources of exposure. The final analyses were completed on March 20, 2025.

**Exposure:**

Social network exposure to CSE among youths.

**Main Outcomes and Measures:**

Experiencing CSE was the main outcome. Logistic regression was used to assess associations between youth network exposure to CSE and the risk of experiencing CSE, and social network analyses were conducted to identify patterns in the connections between youths with and without CSE experiences.

**Results:**

A total of 997 youth were included in the study (mean [SD] age, 14.7 [2.1] years; 903 female [90.6%]). Youths exposed to CSE through their social networks were significantly more likely to experience CSE (adjusted odds ratio, 2.92; 95% CI, 1.91-4.47). A significant interaction between social exposure and childhood adversities revealed that social network exposure was associated with higher CSE risk among youth with fewer adversities (adjusted odds ratio, 1.45; 95% CI, 1.22-1.74). Network analyses revealed that youth experiencing CSE were more likely to be connected to one another (β [SE], 0.32 [0.14]), highlighting the clustering of CSE within social networks.

**Conclusions and Relevance:**

These findings suggest that prevention strategies should aim to reduce unmitigated exposure to individuals experiencing CSE and focus on fostering supportive and healthy relationships. This study underscores the potential for social network modeling to identify at-risk youth and inform prevention efforts.

## Key Points

**Question:**

What is the association between social network exposure to commercial sexual exploitation (CSE) and risk of youths experiencing CSE?

**Findings:**

In this cross-sectional study of 997 youths, network exposure was significantly associated with a higher risk of CSE, especially among youths with fewer childhood adversities. Network analysis showed that youths with CSE experiences connected more often with others who shared similar experiences.

**Meaning:**

These findings suggest that reducing social exposure to high-risk networks may help prevent CSE and that network modeling may identify at-risk youths and inform prevention efforts.

## Teaser

This cross-sectional study examines the association between social network exposure to commercial sexual exploitation (CSE) and youth risk of experiencing CSE, focusing on the interaction between exposure and childhood adversities.

## Introduction

Commercial sexual exploitation (CSE) of children is a public health concern with profound deleterious effects on an individual’s health and well-being, including posttraumatic stress disorder, anxiety, and depression; sexually transmitted infections; unwanted pregnancy; and other physical and mental health problems.^1,2,3^ Defined as the sexual abuse of children for economic gain or other benefits through activities such as commercial sex, pornography, or other forms of sexual exploitation, CSE represents a crucial concern within systems such as child welfare.

While efforts to address CSE have traditionally been reactive, with a focus on identifying and punishing offenders,^4,5^ recently, scholars have advocated for preventive approaches focused on averting targeted harmful behaviors by identifying what puts young people (aged 6-26 years) at risk of CSE.^6,7^ In practice, the Preventing Sex Trafficking and Strengthening Families Act of 2014 (title IV-E, §101) mandates child welfare practitioners to develop protocols for identifying and assisting children at risk of sex trafficking. However, for prevention, identification, and early-intervention efforts to be effective, more research on CSE risk is needed.

Considerable empirical efforts have been made to identify a range of factors associated with CSE risk, which has informed the development of screening and assessment tools aimed at identifying vulnerable youth.^8,9,10,11,12^ However, traditional CSE risk research has been criticized for its narrow focus on individual and, to some extent, familial determinants, such as histories of abuse, neglect, and familial instability,^8,9,10,11,12^ which may perpetuate narratives that marginalize those who experienced harm.^9,13,14^ Moreover, this perspective overlooks broader systemic influences and the complex interplay of social, economic, and community-level factors associated with CSE vulnerability.^15,16^

Therefore, recent scholarship has advocated for a socioecologic understanding of CSE risk to extend beyond individual-level risk factors and recognize how systemic social and community-level dynamics may contribute to vulnerability. Accordingly, empirical research has begun to include risks and vulnerabilities at other levels, including at the interpersonal level (eg, exposure to CSE within social networks), community level (eg, neighborhood disadvantage, social marginalization, systemic inequities), and societal level (eg, gender-based discrimination and violence).^6,16^ This shift in focus necessitates community-wide awareness and prevention strategies that go beyond traditional individualized interventions and address risks and vulnerabilities in the socioecologic surroundings of young people.^6,7^

The role of social networks, particularly, is pivotal in the lives of youths who often begin developing more connections outside of the family context. Although social networks have been extensively examined in the context of other forms of crime and exposure to harmful behaviors,^17,18,19^ the impact of social networks on CSE risk has been underexplored. Exceptions in the literature highlight the dual role of family, peer, and other social networks as both risk and protective factors. For example, previous qualitative work has suggested how both family and peers influence and mitigate risk, with some youths being introduced to exploitation through friends and family members^20,21,22^ while others find themselves in supportive, trusting, and safe relationships that help prevent exploitation.^20,22,23^ In addition, several quantitative studies have supported that social proximity to other youths engaged in CSE or involved in general delinquent behaviors may heighten CSE risk.^16,24^ These studies have collectively underscored the importance of social networks as sources of potential risk or support, yet rigorous quantitative work examining the extent and impact of social exposure to CSE is lacking.

The aim of this study is to help bridge this gap by examining the association of social exposure to CSE with risk of experiencing CSE among youths. Secondly, we leveraged social network analyses and network simulations to address social clustering among youths with CSE experiences to quantify the need of improved data on the interpersonal relationships of youths at risk of CSE.

## Methods

This cross-sectional study used data on youths referred to a specialized CSE program at a children’s advocacy center in the northeastern US between January 1, 2015, and December 31, 2022. The data collection and analyses were approved by the Northeastern University Institutional Review Board with a waiver of youth assent or parental consent as deidentified administrative data were received for this study. The study followed the Strengthening the Reporting of Observational Studies in Epidemiology (STROBE) reporting guideline.

Referrals came primarily from child welfare and criminal justice agencies, indicating concerns that these youths were at risk of or suspected to have experienced CSE. Our study’s sample, therefore, focused on a high-risk group rather than a general population, aligning with previous studies using similar samples.^24,25,26^ Most youths had a history of childhood adversities, consistent with prior research.^8,9,10^ Upon referral, detailed information about the youths, including demographic data, experiences of adversity, and social network contacts (eg, family members, alleged offenders, potential contacts with other youths), was systematically recorded in a case management system.

### Measures

#### CSE Experience

This study’s primary outcome measure was confirmed CSE involvement (with 1 = yes) based on legal criteria and representing varying identification procedures. Such criteria and procedures included agency discovery, youth disclosure, observed or reported engagement in survival sex, or being identified in advertisements regarding sexual exchange.

#### Social Exposure to CSE

The main independent variable concerns whether youths were exposed to CSE through their interpersonal relationships (1 = yes), which was determined in 2 steps. First, case coordinators assessed whether youths reported spending time with people known to be involved in CSE (without reporting the specific contacts).

**Figure 1.**
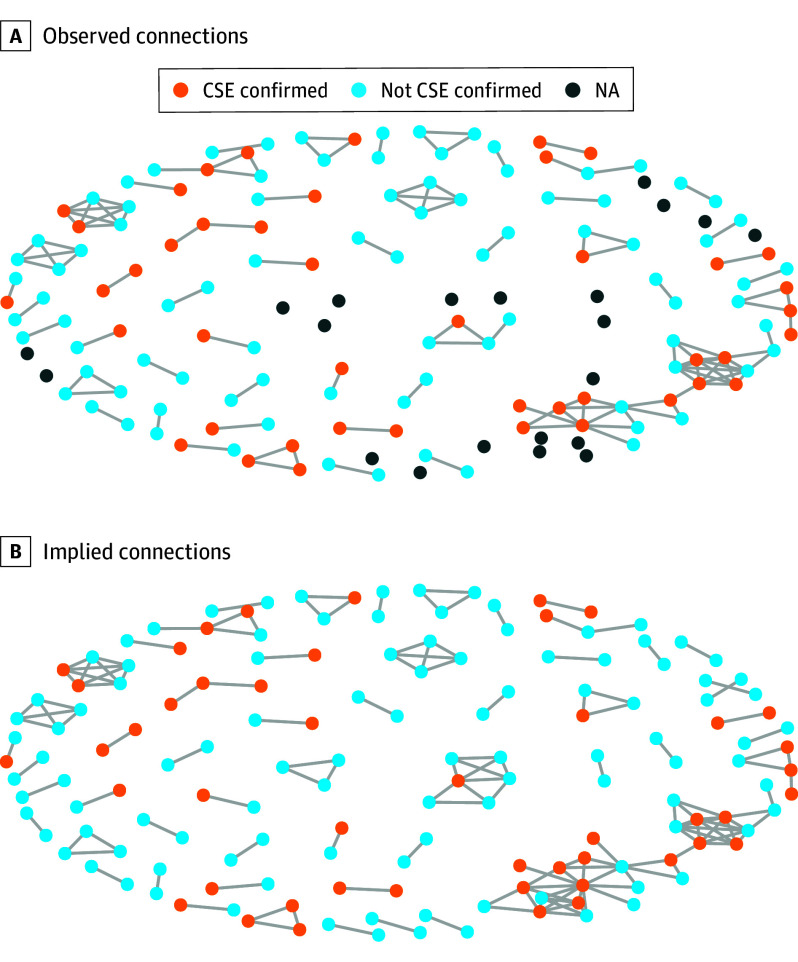
Observed and Implied Networks of Commercial Sexual Exploitation (CSE) Connections Shown are reported social network contacts for a subsample of referred youths (n = 131). Examined are youths who were connected to other referred youths, which was observed when youths had the same alleged offender or were acquaintances or friends and implied for youths who resided in the same residential location within a time frame of 60 days. NA indicates not applicable.

Second, using the reported social network contacts for a subsample of referred youths, we examined which youths were connected to other referred youths, which was observed when youths had the same alleged offender or were acquaintances or friends and implied for youths who resided in the same residential location within a time frame of 60 days (Figure 1). The specific residential placements were determined based on information about groups of youths in a specific zip code. A period of 60 days was chosen as a period long enough for youths at risk of CSE being in residential placement but short enough to not overestimate ties.

#### Other Covariates

We included various covariates, identified in previous research, that may influence CSE risk.^8,9,10,11,12^ First, to account for the role of having experienced previous and/or current adversities, we created the following measures: (1) personal childhood adversities, which we included as a sum of having experienced any type of abuse and a history of being missing or on the run, mental health concerns, and substance use; (2) familial adversities, which we calculated as the sum of variables indicating whether youths had witnessed family separation and abuse, substance use, and homelessness at the family level; (3) violence, which we calculated as the sum of youths having received threats, physical assaults, or sexual assaults or being in an abusive relationship; (4) proximal risks, which we included as the sum of having been seen in CSE areas, having knowledge of CSE rumors, or having been previously approached for engaging in CSE; (5) mental health concerns at the time of referral as reported at intake by the children’s advocacy center (eg, attention-deficit/hyperactivity disorder; posttraumatic stress disorder; anxiety, bipolar, conduct, mood, oppositional defiant, or panic disorder; depression; suicidal ideation or suicide attempts; self-harming behaviors; schizophrenia), included as a dichotomous variable (1 = yes); and (6) missing or on the run at the time of referral, included as a dichotomous variable (1 = yes). Furthermore, we accounted for child welfare involvement (1 = yes) and criminal justice system involvement (1 = yes) before or at the time of referral. Finally, we accounted for the potential role of personal characteristics, including gender (1 = female), age, and being referred since the start of the COVID-19 pandemic (March 11, 2020), which have been reported to have had an impact on CSE risk, identification, and services.^25^ Gender identities were reported in the dataset, but because of the relatively small sample sizes, youths identifying as male or transgender were combined into a reference category in the analyses. Age was imputed for 40 youths using predictive mean matching via the mice package in R, version 4.3.0 (R Foundation for Statistical Computing), which imputes missing age values based on similar observed cases.^27^ Race and ethnicity were excluded due to 25% missing data (n = 249). Sensitivity analyses in the reduced sample, using Black and White as covariates (other races and ethnicities as the reference), yielded similar main findings. Categories followed the children’s agency center definitions. However, an interaction between social exposure and childhood adversities (see Results) was no longer significant, which may have been due to case loss. The exponential random graph models (ERGMs) (see Statistical Analysis) subsample had no missing race and ethnicity data.

### Statistical Analysis

We conducted 2 types of analyses. First, logistic regression was used to examine the association between social exposure to CSE and experience of CSE, which is appropriate when the outcome variable is dichotomous. Statistical significance was set at *P* < .05. To explore whether the role of social exposure varied across different groups, we included 2-way interactions between CSE exposure and other covariates, such as childhood adversities. Multicollinearity was not a concern (mean [SD] variance inflation factor, 1.15 [0.13]).

Second, ERGMs were applied to assess the structural patterns of social connections among a subsample of referred youths for whom specific social connections were reported, examining whether youths who experienced CSE were more likely to be connected to one another. While logistic regression establishes the individual-level association between social exposure to CSE and risk of experiencing CSE, ERGMs provide insight into whether social clustering of youths with CSE experiences is structured beyond what would be expected by chance, accounting for broader network dependencies. Specifically, ERGMs allow for the analysis of node-level characteristics (eg, CSE experience), edge-level characteristics (eg, shared experiences of childhood adversities), and network characteristics (eg, tendencies toward triad or dyad formation assessed by so-called geometrically weighted edge-shared partners and geometrically weighted dyad-shared partners, respectively). These models compare observed networks with random networks, using maximum pseudo-likelihood estimations and Markov chain Monte Carlo methods for estimation.^28,29,30^ All data processing and analyses were conducted in R, version 4.3.0 using the statnet suite of packages for ERGMs.^31^ The final analyses were completed on March 20, 2025.

## Results

### Social Exposure to CSE and Experience of CSE

The results are based on 997 youths, the majority of whom were female (903 [90.6%] vs 90 male or transgender youths [9.4%]) with a mean (SD) age of 14.7 (2.2) years (Table 1). Of the total sample, 217 youths (21.8%) were confirmed to have experienced CSE across any of their referrals to the agency (270 youths [27.1%] were referred multiple times); 134 (13.4%) reported spending time with people known to be involved in CSE; and 69 (6.9%) had ties to others involved in CSE, such as shared offenders or residence in the same facility. Combining all measures of social exposure, 172 youths (17.3%) were exposed to CSE through their social networks (specific breakdowns of types of connections provided in the eTable and eFigure 1 in Supplement 1).

**Table 1.** Descriptive Statistics (N = 997)

Characteristic	Youths, No. (%)
CSE confirmed	217 (21.8)
Social exposure	172 (17.3)
Adversities	
History of personal childhood adversities, mean (SD) [range]	1.6 (1.2) [0-4.0]
History of familial adversities, mean (SD) [range]	0.9 (1.0) [0-4.0]
Referral for violence, mean (SD) [range]	0.5 (0.8) [0-4.0]
Referral for CSE proximal risks, mean (SD) [range]	0.4 (0.7) [0-3.0]
Referral for mental health concerns	345 (34.6)
Missing referral data	293 (29.4)
System involvement	
Child welfare involvement	935 (93.8)
Criminal justice involvement	586 (58.8)
Personal characteristics	
Sex	
Female	903 (90.6)
Male or transgender^a^	94 (9.4)
Age, mean (SD) [range], y	14.7 (2.1) [6.0-26.0]^b^
Post–COVID-19 referral	412 (41.3)

Abbreviation: CSE, commercial sexual exploitation.

^a^ Due to the relatively small sample size, male youths (n = 80) and youths identifying as transgender (n = 14) were combined into a reference category in the analyses.

^b^ Median (IQR), 15.0 (13.0-16.0) years.

Logistic regression analyses revealed that social exposure to CSE significantly increased the likelihood of personal CSE experience (Table 2). Of all risk factors, social exposure to CSE was associated with the highest CSE risk (adjusted odds ratio [AOR], 2.92; 95% CI, 1.91-4.47; *P* < .001). Other significant risk factors included personal childhood adversities (AOR, 1.45; 95% CI, 1.22-1.74; *P* < .001), proximal risks such as being seen in areas known for CSE (AOR, 1.93; 95% CI, 1.52-2.44; *P* < .001), and experiences of violence reported at the time of referral (AOR, 1.58; 95% CI, 1.31-1.92). The likelihood of experiencing CSE decreased among youths with prior or current child welfare involvement (AOR, 0.36; 95% CI, 0.19-0.70; *P* = .002), referred since the onset of the COVID-19 pandemic (AOR, 0.44; 95% CI, 0.30-0.64; *P* < .001), and who identified as female (AOR, 0.53; 95% CI, 0.31-0.95; *P* = .03).

**Table 2.** Results From Logistic Regression Analyses

Variable	Model 1^a^		Model 2^b^	
	β (SE)	AOR (95% CI)	β (SE)	AOR (95% CI)
Social exposure	1.07 (0.22)^c^	2.92 (1.91-4.47)	1.79 (0.40)^c^	6.00 (0.27-13.10)
Interaction: personal childhood adversities by social exposure	NA	NA	−0.37 (0.17)^d^	0.69 (0.50-0.97)
Adversities				
Personal childhood adversities	0.37 (0.09)^c^	1.45 (1.22-1.74)	0.47 (0.10)^c^	1.60 (1.31-1.96)
Familial adversities	−0.11 (0.09)	0.89 (0.74-1.07)	−0.10 (0.09)	0.90 (0.75-1.08)
CSE proximal risks	0.66 (0.12)^c^	1.93 (1.52-2.44)	0.63 (0.12)^c^	1.87 (1.48-2.38)
Violence	0.46 (0.10)^c^	1.58 (1.31-1.92)	0.47 (0.10)^c^	1.60 (1.33-1.94)
Mental health concerns	0.19 (0.20)	1.21 (1.82-1.79)	0.17 (0.20)	1.19 (0.80-1.75)
Missing	0.02 (0.19)	1.02 (0.70-1.49)	0.02 (0.19)	1.02 (0.69-1.48)
System involvement				
Child welfare involvement	−1.03 (0.34)^e^	0.36 (0.19-0.70)	−1.08 (0.35)^e^	0.34 (0.17-0.68)
Criminal justice involvement	0.04 (0.18)	1.04 (0.73-1.50)	0.07 (0.19)	1.07 (0.75-1.54)
Personal characteristics				
Female	−0.63 (0.29)^d^	0.53 (0.31-0.95)	−0.65 (0.29)^d^	0.52 (0.30-0.94)
Age	−0.01 (0.05)	1.00 (0.91-1.09)	−0.01 (0.05)	0.99 (0.91-1.09)
Post–COVID-19 referral	−0.82 (0.19)^c^	0.44 (0.30-0.64)	−0.83 (0.19)^c^	0.43 (0.29-0.63)
Intercept	−0.91 (0.79)	0.40 (0.08-1.85)	−1.02 (0.79)	0.36 (0.07-1.68)
No.	997	NA	997	NA
Log-likelihood	−419.20 (*df* = 13)	NA	−416.93 (*df* = 14)	NA
Pseudo *R*^2^	0.20	NA	0.20	NA

Abbreviations: AOR, adjusted odds ratio; CSE, commercial sexual exploitation; NA, not applicable.

^a^ Base model.

^b^ Interaction effect between social exposure and personal childhood adversities added.

^c^ *P* < .001.

^d^ *P* < .05.

^e^ *P* < .01.

**Figure 2.**
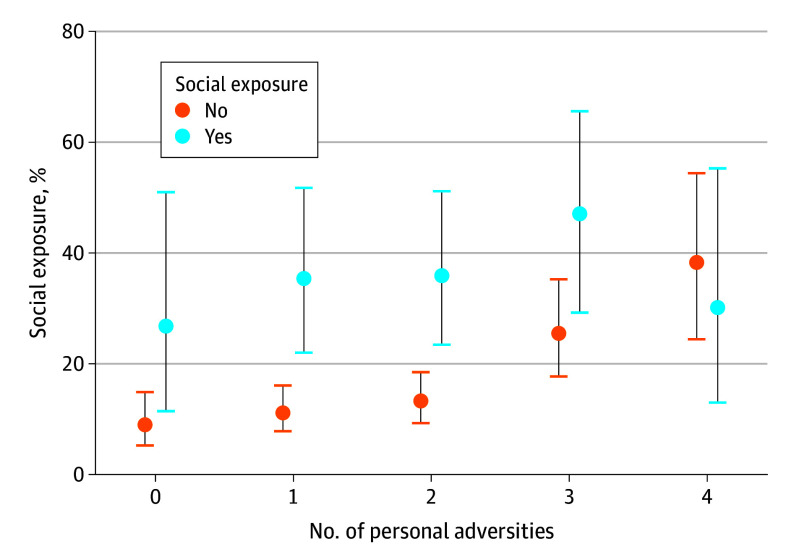
Marginal Probabilities for the Interaction of Personal Childhood Adversities by Social Exposure Social exposure is a binary measure (0 = not exposed; 1 = exposed). Refer to model 2 in Table 2. Error bars indicate the 95% CIs.

Moreover, we found a significant negative interaction between social exposure to CSE and previous childhood adversities (model 2 in Table 2). As illustrated in Figure 2, this interaction means that social exposure was associated with higher CSE risk most substantially among youths with fewer adversities (AOR, 0.69; 95% CI, 0.50-0.97; *P* = .03).

### Social Clustering of Youths With CSE Experiences

The harmful association of social exposure to CSE with the risk of personal experience of CSE highlights the need to understand the mechanisms behind the connections between at-risk youths. Therefore, we used ERGMs to identify whether the reported networks resembled a process of social clustering of youths with CSE experiences. While some network characteristics (eg, small and low-density networks) can limit goodness-of-fit for some metrics (eFigure 3 in Supplement 1), the analysis provided valuable insights into social tie dynamics. The base model (Table 3) showed that youths with CSE histories were less likely to be connected with peers without CSE experiences (β [SE], −0.62 [0.13]; *P* < .001) but were more likely to be connected with one another (β [SE], 0.32 [0.14]; *P* = .03). This finding suggests the importance of homophily based on shared CSE experiences. Furthermore, the networks displayed a preference for clustering in triads (3 paths) rather than dyads (2 paths), as indicated by positive geometrically weighted edge-shared partner and negative geometrically weighted dyad-shared partner terms, respectively. Triads are often considered a more stable form of social interaction and trust, and the experiences and behaviors of one’s triad members may have a more profound impact on personal experiences and behaviors.

**Table 3.** Results (Logit Probabilities) From the Exponential Random Graph Models

Logit probability	β (SE)
Edges	−2.12 (0.44)^a^
Node level	
CSE indicated	−0.62 (0.13)^a^
Edge level	
CSE homophily	0.32 (0.14)^b^
Network level	
GWDSP (decay parameter *r* = 0.15)	−0.96 (0.11)^a^
GWESP (decay parameter *r* = 0.15)	2.21 (0.14)^a^

Abbreviations: CSE, commercial sexual exploitation; GWDSP, geometrically weighted dyad-shared partners; GWESP, geometrically weighted edge-shared partners.

^a^ *P* < .001.

^b^ *P* < .05.

Simulations based on ERGM estimates revealed that for 172 youths exposed to CSE, network density closely matched observed data (simulated density *G* = 0.011). According to this simulated network, 96 of these youths (55.8%) had exposure to CSE through social ties reported to child welfare (eFigure 2 in Supplement 1). These findings may inform research on indirect social exposure and aid in developing strategies to protect youths from CSE.

## Discussion

The purpose of this cross-sectional study was to assess the importance of social exposure to CSE for increasing personal risk of experiencing CSE. Our findings support 3 main conclusions. First, youths with social exposure to CSE were nearly 3 times more likely to experience CSE than those without such exposure. Social exposure was the strongest risk factor, exceeding childhood adversities, proximal risks, and experiences of other types of violence. These findings emphasize the importance of social proximity in amplifying CSE risk, particularly through familial and peer relationships.^16,20,21,24^

Second, a key finding was that youths with fewer adversities but exposed to CSE through social networks were as vulnerable as those with extensive histories of adversities. One possible explanation for this pattern is that youths with extensive childhood adversities already face a heightened baseline risk of experiencing CSE due to cumulative vulnerabilities, including prior system involvement and unstable living situations. In contrast, for youths with fewer childhood adversities, social exposure may be a more influential factor in increasing their risk. This finding adds to previous research by highlighting how even youths without prior adversities may face high CSE risk due to social connections, offering insight into an often-overlooked population in the existing literature on CSE.^24,32^

Third, network analyses revealed that youths with histories of CSE experience were more likely to be connected with one another. This finding aligns with previous research highlighting the role of homophily in shared harmful experiences.^19,33^ Homophily in CSE networks may stem from social distrust toward others while feeling understood and less stigmatized by peers with shared experiences.^34,35^

### Clinical Implications

Together, these findings reinforce the need for public health strategies that go beyond individual-level interventions and include community-based and structural efforts to reduce exploitation risks. These efforts include CSE prevention, early identification, and reduction of its long-term impact.

#### Primary Prevention: Preventing CSE

Our findings emphasize the need for programs that reduce youth exposure to high-risk peer networks and support healthy relationships, especially for youths without prior CSE experience. Congregate care placements may unintentionally increase exposure to CSE. Research has shown that youths in these settings often have histories of exploitation, being missing from care, or being vulnerable to recruitment. Some traffickers exploit peer-to-peer relationships to entrap new recruits.^20,21,22^

#### Secondary Prevention: Early Identification of CSE

Youths connected to peers involved in commercial sex should signal to practitioners a high risk of CSE. Screening tools should incorporate social network factors alongside traditional risk markers, such as childhood adversities.^8,9,10,11,12^ Our study highlights the need to assess social exposure risks, even for youths without histories of childhood adversity. Practitioners should monitor social connections and support protective relationships.

#### Tertiary Prevention: Reducing Long-Term Impact

While network exposure to CSE is associated with increased risk, social networks may also aid recovery. Programs helping people with CSE experiences build healthy relationships with peers who have exited exploitation have shown promise in reducing future exploitation and improving well-being. Early evidence has suggested that these interventions help break cycles of exploitation.^36^

### Limitations

Several limitations temper our conclusions. First, shortfalls of administrative data may result in incomplete information on social connections, although we have mitigated this challenge as much as possible by combining 2 measures gauging social exposure to CSE in our main analyses. Second, missing information on important covariates, especially related to race and ethnicity, may have resulted in underspecification of our models. We urge future studies to replicate our analyses across different racial and ethnic groups. Third, ERGMs require further verification, especially because of incomplete information on the specific ties between youths at risk of CSE. More complete networks could guide further analyses on social network processes associated with CSE and may enhance the identification of CSE exposure. Fourth, because our data are cross-sectional, we cannot definitively determine whether social exposure to CSE preceded or followed an individual’s own experience of CSE. Some youth may have experienced CSE prior to forming social ties with other CSE-experienced peers, while others may have been influenced by these relationships. Longitudinal research is needed to disentangle these temporal dynamics and better understand causal pathways.

## Conclusions

In this cross-sectional study of 997 youth, social exposure was significantly associated with an increased risk of experiencing CSE. The findings underscore the public health urgency of addressing social exposure to CSE, as such exposure was associated with an increased risk of CSE experience. By minimizing harmful connections and fostering supportive relationships, practitioners could help disrupt pathways to victimization and mitigate the long-term health consequences of CSE, including heightened risks for trauma-related disorders, sexually transmitted infections, and adverse social outcomes. While this study’s aim was to guide research in new directions by unraveling which social relations exist and whether exposure matters, further research is needed to deepen our understanding of what drives social exposure and to develop tailored interventions for diverse populations.

## Notes

Supplement 1. **eTable. **Comparison of Social Exposure to CSE Based on Observed Versus Implied Connections **eFigure 1. **Observed and Implied Connections Among Youths at Risk of CSE **eFigure 2.** Simulated CSEC Network **eFigure 3. **Goodness-of-Fit Indices for ERGM

Supplement 2.
Data Sharing Statement
