# Supplementary material for: Social Network Exposure to Commercial Sexual Exploitation and Risk of Harm to Youths
Source: JAMA Netw Open. 2025 Jun 10;8(6):e2513520. doi: 10.1001/jamanetworkopen.2025.13520 (PMC12152702; doi:10.1001/jamanetworkopen.2025.13520)
Supplement: Supplement 1. — eTable. Comparison of Social Exposure to CSE Based on Observed Versus Implied Connections eFigure 1. Observed and Implied Connections Among Youths at Risk of CSE eFigure 2. Simulated CSEC Network eFigure 3. Goodness-of-Fit Indices for ERGM [file jamanetwopen-e2513520-s001.pdf]

Supplemental Online Content

de Vries I, Kafafian M, Gobar S, Farrell A. Social network exposure to commercial sexual exploitation and risk of harm to youths. *JAMA Netw Open*. 2025;8(6):e2513520. doi:10.1001/jamanetworkopen.2025.13520

eTable. Comparison of Social Exposure to CSE Based on Observed Versus Implied Connections

eFigure 1. Observed and Implied Connections Among Youths at Risk of CSE

eFigure 2. Simulated CSEC Network

eFigure 3. Goodness-of-Fit Indices for ERGM

This supplemental material has been provided by the authors to give readers additional information about their work.

© 2025 de Vries I et al. *JAMA Network Open*.

eTable. Comparison of Social Exposure to CSE Based on Observed Versus Implied Connections

A) Observed Connections and Agency Registration on General Connections with CSE-involved People [a]

	No Agency Registration of CSE-involved people	Agency Registration of CSE-involved people
No Observed Connections	831	105
Observed Connections	32	29

Note. *166 youths were exposed to CSE through their social network.

B) Observed and Implied[b] *Connections and Agency Registration on General Connections with CSE-involved People.*

	No Agency Registration of CSE-involved people	Agency Registration of CSE-involved people
No Observed Connections	825	103
Observed Connections	38	31

Note. [a] 172 youths were exposed to CSE through their social network. [b] based on shared residential placement within a 60-day time window

eFigure 1. Observed and Implied Connections Among Youths at Risk of CSE

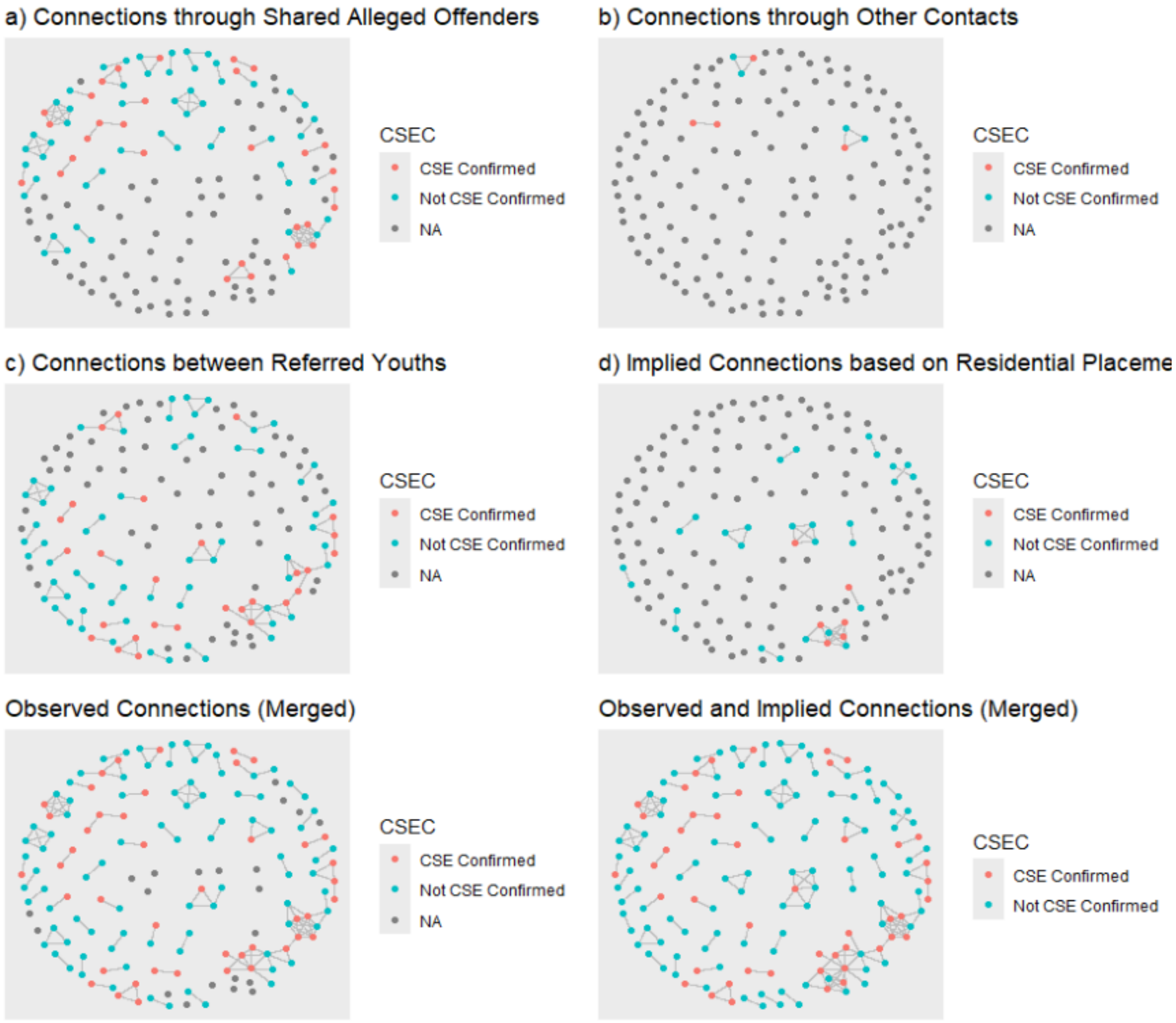

© 2025 de Vries I et al. *JAMA Network Open*.

eFigure 2. Simulated CSEC Network (N = 172)

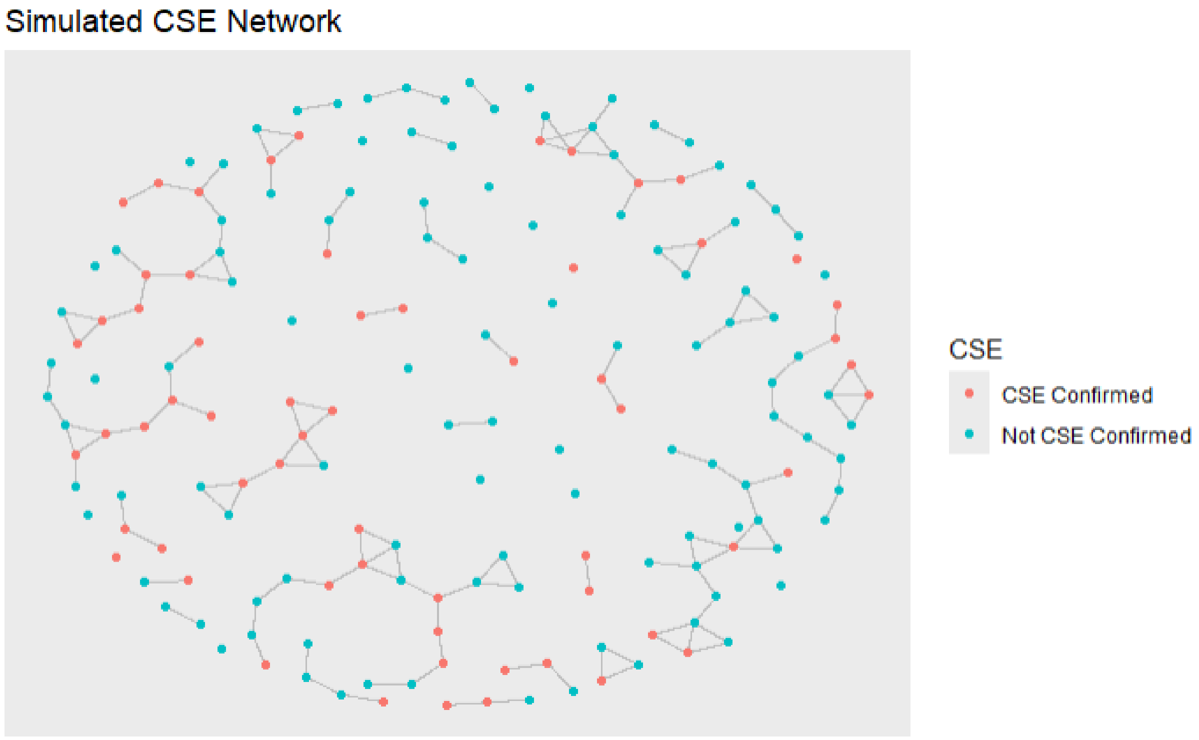

© 2025 de Vries I et al. *JAMA Network Open*.

eFigure 3. Goodness-of-Fit Indices for ERGM

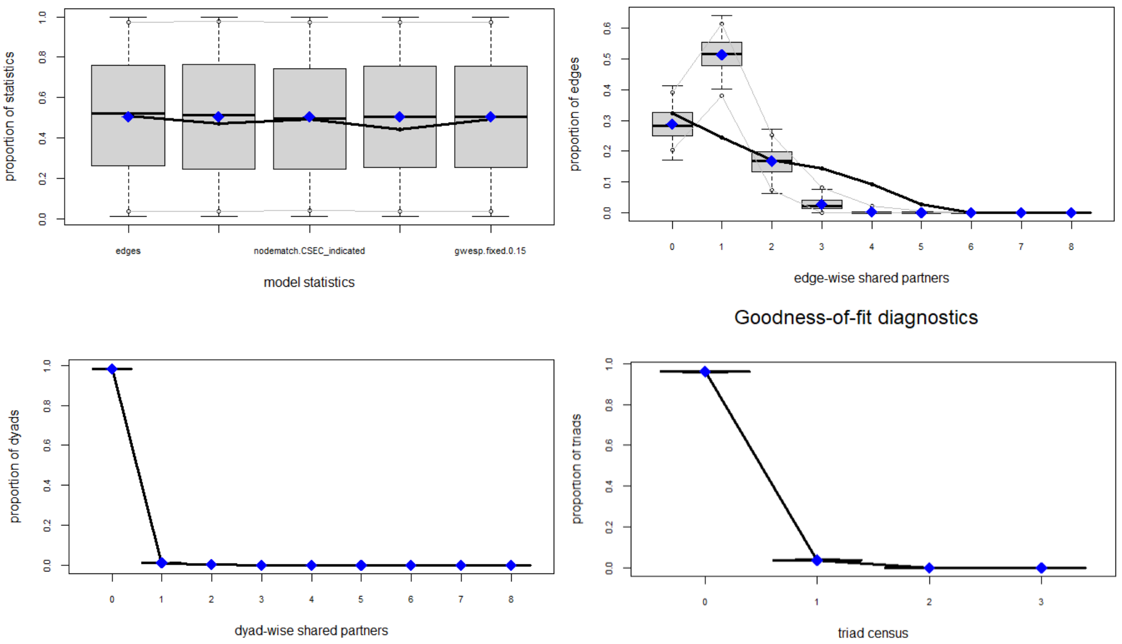

© 2025 de Vries I et al. *JAMA Network Open*.
