# Supplementary material for: Social Network Exposure to Commercial Sexual Exploitation and Risk of Harm to Youths
Source: JAMA Netw Open. 2025 Jun 10;8(6):e2513520. doi: 10.1001/jamanetworkopen.2025.13520 (PMC12152702; doi:10.1001/jamanetworkopen.2025.13520)
Supplement: Supplement 2. — Data Sharing Statement [file jamanetwopen-e2513520-s002.pdf]

Data Sharing Statement

de Vries. Social Network Exposure to Commercial Sexual Exploitation and Risk of Harm to Youths. *JAMA Netw Open*. Published June 10, 2025.
doi:10.1001/jamanetworkopen.2025.13520

Data
Data available: No

Additional Information
Explanation for why data not available: These data are highly sensitive as they include information about minors who were sexually exploited and characteristics about their personal, health and socioecological context. Should researchers want to replicate our study, we can share our code and facilitate access through the relevant institutions.
